# MiR-145 functions as a tumor suppressor via regulating angiopoietin-2 in pancreatic cancer cells

**DOI:** 10.1186/s12935-016-0331-4

**Published:** 2016-08-25

**Authors:** Hao Wang, Cheng Hang, Xi-Long Ou, Jin-Shan Nie, Yi-Tao Ding, Shi-Gui Xue, Hua Gao, Jian-Xin Zhu

**Affiliations:** 1 Department of General Surgery, DrumTower Clinical Medical College of Nanjing Medical University, Nanjing, 210008 Jiangsu Province China; 2 Department of Gastroenterology, The First People’s Hospital of Taicang, Suzhou, 215400 Jiangsu Province China; 3 Department of Gastroenterology, Zhongda Hospital, Southeast University, Nanjing, 210009 Jiangsu Province China

**Keywords:** miR-145, Ang-2, Pancreatic cancer, Angiogenesis

## Abstract

**Background:**

Pancreatic cancer is currently one of the leading causes of cancer deaths without any effective therapies. Mir-145 has been found to be tumor-suppressive in various types of cancers. The aim of this study is to investigate the role of miR-145 in pancreatic cancer cells and explore its underlying mechanism.

**Methods:**

Quantitative real time PCR was used to determine the expression level of miR-145 and angiopoietin-2 (Ang-2) mNRA, and the expression level of Ang-2 protein was measured by western blotting. The anti-cancer activities of miR-145 were tested both in in vitro by using cell invasion and colony formation assay and in vivo by using xenograft assay. The direct action of miR-145 on Ang-2 was predicted by TargetScan and confirmed by luciferase report assay. The vascularization of xenografts were performed by immunohistochemical analysis.

**Results:**

The expression level of miR-145 was significantly lower and the expression levels of Ang-2 mRNA and protein was significantly higher in the more aggressive pancreatic cancer cells (MiaPaCa-2 and Panc-1) when compared to that in BxPC3 cells. Overexpression of miR-145 in the BxPC3, MiaPaCa-2 and Panc-1 cells suppressed the cell invasion and colony formation ability, and the expression level of Ang-2 protein in MiaPaCa-2 and Panc-1 cells was also suppressed after pre-miR-145 transfection. Intratumoral delivery of miR-145 inhibited the growth of pancreatic cancer xenografts and angiogenesis in vivo, and also suppressed the expression level of angiopoietin-2 protein. Luciferase report assay showed that Ang-2 is a direct target of miR-145, and down-regulation of angiopoietin-2 by treatment with Ang-2 siRNA in the BxPC3, MiaPaCa-2 and Panc-1 cells suppressed cell invasion and colony formation ability. The reverse transcription PCR results also showed that Tie1 and Tie2 were expressed in BxPC3, MiaPaCa-2 and Panc-1 cells.

**Conclusion:**

MiR-145 functions as a tumor suppressor in pancreatic cancer cells by targeting Ang-2 for translation repression and thus suppresses pancreatic cancer cell invasion and growth, which suggests that restoring of miR-145 may be a potential therapeutic target for pancreatic cancer.

## Background

Pancreatic cancer was the fourth leading cause of cancer-related deaths and more than 45,000 new cases were estimated in 2013 [1]. Recent studies showed that tumor suppressor loci were mutated or down-regulated in human pancreatic tumors, which accelerated tumor progression and resulted in invasive and metastatic malignancies [2]. However, the role of tumor suppressors in pancreatic tumors are still largely unknown.

MicroRNAs (miRNAs) are a family of non-coding RNAs with a short length of 19–25 nucleotides. MiRNAs functioned as oncogene or tumour suppressor by binding to the 3′-untranslated region (UTR) of target genes to regulate their expression [3–5]. MiRNAs play an important role in many physiological and pathological processes, including in almost all aspects of cancer biology, such as cell proliferation, apoptosis, invasion/metastasis, and angiogenesis [6]. Studies have shown that miR-145 has important implications in etiology, treatment and pathogenesis of cancer, including pancreatic cancers. Previous studies demonstrated the tumor suppressive role of miR-145 in caners, in which miR-145 suppressed liver and head and neck cancer cell invasion by targeting on ADAM metallopeptidase domain 17 [7, 8]. MiR-145 was also reported to repress pluripotency in human embryonic stem cells via regulating the expression of octamer-binding transcription factor 4, sex determining region Y-box 2 and Kruppel-like factor 4 [9]. Moreover, in many other types of cancers, miR-145 and its direct target were also reported, for example, miR-145 directly targets AKT3 in thyroid cancer [10], and miR-145 also targets Mucin 1, cell surface associated in metastatic breast cancer [11], p70S6K1 in colon cancer [12], insulin-like growth factor receptor 1 in human bladder cancer cells [13], c-Myc in non-small cell lung cancer [14] and the transcription factor signal transducer and activator of transcription 1 in colon cancer [15]. Recently, Khan et al. [16] reported that miR-145 targeted Mucin 13, cell surface associated to suppress growth and invasion of pancreatic cancer cells.

Angiopoietin-2 (Ang-2) is the ligand for an endothelial cell-specific tyrosine kinase receptor, and plays a key role in angiogenesis and tumor progression [17, 18]. Previous study reported that Ang-2 played a significant role in pancreatic carcinoma angiogenesis, and knockdown of Ang-2 induced anti-angiogenesis effect both in vitro and in vivo [19, 20]. Up to date, there is no evidence showing that expression of Ang-2 is linked with miRNAs in pancreatic cancers. In the present study, by using bioinformatics analytic tool (Targetscan), the 3′UTR of Ang-2 gene was found to be a target of miR-145. Here, we documented the tumor suppressive role of miR-145 in pancreatic cell lines. Subsequent analyses further established the relationship between miR-145 and Ang-2 in pancreatic cancer cells.

## Methods

### Cell culture

The human pancreatic cancer cells (MiaPaCa-2 and Panc-1) were cultured in Dulbecco’s Modified Eagle’s Medium (DMEM, Life Technologies, Inc., Gaithersburg, MD) and BxPC-3 cells were cultured in RPMI-1640 medium (Life Technologies, Inc., Gaithersburg, MD), and both medium were supplemented with 10 % FBS (Life Technologies, Inc.) and 100 units/ml penicillin and 100 units/ml streptomycin. The BxPC-3, MiaPaCa-2 and Panc-1 cells were seeded to be 60–80 % confluent in 6-well plates 24 h before the cells were transfected with 30 pmol of either precursor of miR-145 (pre-miR-145; Ambion; P/N: AM17100, Product ID: PM11480) or scramble miRNA (Pre-miR™ miRNA Precursor Negative Control #1, P/N: AM17110); and angiopoietin-2 siRNA (siAng-2) or scramble (RiboBio, Guangzhou) using the Lipofectamine RNAiMAX reagent (Invitrogen, Carlsbad, CA) according to the manufacturer’s instructions. Forty-eight hours after transfection, cells were processed for further experiments as described below.

### In vitro invasion assay

The invasion assays in the pancreatic cancer cell lines were performed in a modified two-chamber assay. 2 × 10^5^ cells were seeded on the upper chamber of 6-well Transwell plates (Costar; Cambridge, MA) coated with Matrigel (Becton–Dickinson, Heidelberg, Germany) diluted at a 1:2 ratio with medium and incubated for 24 h. The lower chamber was filled with DMEM containing 10 % FBS. After 24 h incubation, cells on the upper side of the membrane were wiped off and the membrane was fixed with 4 % paraformaldehyde and 0.25 % glutaraldehyde. Cells on the lower side of the membrane were stained with 0.5 % methylene blue in 50 % methanol and invaded cells were counted under a microscope. All invasion assays were done in triplicate.

### Quantitative real-time PCR (qRT-PCR) analysis

Total RNA was extracted from cultured cells using TRIzol^®^ reagent (Invitrogen). DNaseI-treated RNA was used for first strand cDNA synthesis using M-MLV reverse transcriptase (Promega) and oligo (dT) 15 according to the manufacture’s protocols and 1 μl cDNA samples were used for conventional PCR amplifications. QRT-PCR analysis was performed in a real-time PCR system (StepOne, Applied Biosystems) and the expression levels of Ang-2 were normalized to GAPDH determined by a SYBR Green-based comparative cycle threshold CT method. Real-time PCR primers were: Ang-2-F: 5′-AGA TTT TGG ACC AGA CCA GTG A-3′, Ang-2-R: 5′-GGA TGA TGT GCT TGT CTT CCA T-3′; GAPDH-F: 5′-TGT GGG CAT CAA TGG ATT TGG-3′, GAPDH-R: 5′-ACA CCA TGT ATT CCG GGT CAA T-3′; miR-145-F: 5′-AAG GGA GTC CAG TTT TCC CAG GAA TCC-3′, miR-145-R: 5′-GTC GTA TCC AGT GCA GGG TCC GAG GTA TTC GCA CTG GAT ACG AC-3′; U6-F: 5′-CTC GCT TCG GCA GCA CA-3′, U6-R: 5′-AAC GCT TCA CGA ATT TGC GT-3′.

### Western blotting

The expression of Ang-2 was measured in pancreatic cancer cell lines (BxPC-3, MiaPaCa-2 and Panc-1) or tumor tissue by western blotting. Protein extraction was performed using the extraction buffer containing 10 mM Tris HCl (pH 7.5), 2 M urea, 2 mM EDTA, 2 mM EGTA, and protease inhibitors. Thirty micrograms of protein were loaded onto SDS–polyacrylamide gels, size fractionated on SDS-PAGE gels, and transferred to a nitrocellulose membrane using the semidry technique. The membranes were blocked with 5 % milk powder in TBST for 1 h. Specific monoclonal anti-Ang-2 (ab8452) and monoclonal anti-β-actin (ab3280) primary antibodies (Abcam Biotechnology, Cambridge, MA, USA) were used, and HRP conjugated immunoglobulin was used as a secondary antibody (Jackson ImmunoResearch Laboratories). West Pico Chemiluminescent (Pierce) was used as the substrate to visualize protein bands, which were quantified using densitometry image analysis software (Image Master VDS; Pharmacia Biotech). Normalization was made against β-actin expression.

### ELISA

Ang-2 concentrations in BxPC3, MiaPaCa-2 and Panc-1 cell culture supernatant were determined after transfection with pre-miR-145 for 48 h. Measurements were made with a commercially available enzyme-linked immunosorbent assay (ELISA) (R&D Systems, Minneapolis, USA) according to the manufacturers’ instructions.

### Soft agar assay

Colony formation and cell growth rate in soft agar were tested by plating 2.5 × 10^4^ of BxPC3, MiaPaCa-2 and Panc-1 cells transfected with scramble or pre-miR-145 in 0.4 ml DMEM, supplemented with 100 units/ml penicillin, 100 g/ml streptomycin, 100 g/ml amphotericin B, 3 % FBS, and 0.3 % low melting temperature agarose (Seaplaque) in 12-well plates (6 wells for each) coated with 0.8 ml 0.6 % low melting temperature agarose. Platelet-derived growth factor-bb (50 ng/ml) was added to half of the wells of each type of transfected cells. Colony formation was monitored for 5 days in 37 °C incubator, and colony number was counted under a microscope.

### In vivo study

Female nude mice (4–6 weeks, 18–20 g) were purchased from the Model Animal Research Center of Nanjing University. 5 × 10^6^ of Panc-1 cells (resuspended in 100 μl saline) were injected subcutaneously into the right flank of each mouse. Tumor volumes were determined every 5 days after injection as described previously [21]. Mice with xenografts volume lager than 500 mm^3^ were treated with intratumoral injection of saline twice a week (saline group), scramble (saline plus scramble group) or pre-miR-145 (saline plus pre-miR-145 group) for 4 weeks. Each treatment group has eight animals. At the end of the experiment, mice were sacrificed and tumors were dissected for immunohistochemistry and western blot analysis. All the animal study was carried out in strict accordance with Institutional Animal Ethics Care and Use Committee of the Jiangsu Cancer Hospital (approved number 21040608). All surgery was performed under sodium pentobarbital anesthesia, and efforts were made to minimize suffering.

### Immunohistochemistry

Immunohistochemical analysis of vascularization was performed using the analySIS system and the monoclonal antibody against Factor VIII (ab41186, abcam Biotechnology, Cambridge, MA, USA). Briefly, 5 µm thick FFPE sections were cut, placed on slides coated with 3-triethoxysilylpropylamine (Sigma, St. Louis, Missouri, USA), and then fixed overnight at 37 °C. After deparaffinization in xylene and rehydrating through graded alcohols, the slides were incubated in H_2_O_2_ to block endogenous peroxidase activity. Then sections were incubated with primary monoclonal antibody against Factor VIII (5 µg/ml) at 4 °C overnight. After overnight incubation, the sections were washed with PBS for 5 min × 3 times, and the sections were then incubated with biotinylated goat anti-rabbit immunoglobulin (dilution 1:300; Vector Laboratories, Burlingame, California, USA). The peroxidase activity was visualized by 3,3′-diaminobenzidine tetrahydrochloride. Hematoxylin was used as a counter stain.

The degree of vascularization was measured by the average number of Factor VIII-positive microvessels in three different areas at 200-fold magnification and recorded as microvessel density (MVD). Briefly, the Factor VIII stained sections were initially scanned at low power (100-fold magnification) and the areas having the highest number of microvessels were selected. Subsequently, microvessel counting was performed in three different areas at 200-fold magnification and the mean value was used for further analysis. Any clearly stained endothelial cells or cell clusters were considered as a single countable microvessel, regarding the presence of lumen and large vessels were automatically excluded from the analysis.

### Constructs and luciferase assay

To determine whether Ang-2 is a downstream mediator of miR-145, the entire human Ang-2 3′-untranslated region (UTR) segment was amplified by PCR using mouse genomic DNA as a template. The PCR products were inserted into the p-MIR-report plasmid (Ambion). For luciferase reporter assay, 1 μg of firefly luciferase reporter plasmid, 0.5 μg of β-galactosidase expression vector (Ambion), and equal amount (200 pmol) of pre-miR-145 or scrambled negative control miRNA were transfected into cells in 6-well plates. The β-galactosidase vector was used as a transfection control. Twenty-four hours after transfection, cells were assayed using luciferase assay kits (Promega).

### Statistics

All results were expressed as mean ± SEM from at least three independent experiments. Significance analysis of normal distributed data were performed using two-tail Student’s t test, One-Way ANOVA, or Two-way ANOVA, as appropriate and P values of less than 0.05 were considered statistically significant.

## Results

### The expression of miR-145 is reduced in more aggressive pancreatic cancer cell lines and accompanied with increased expression of Ang-2

**Fig. 1:**
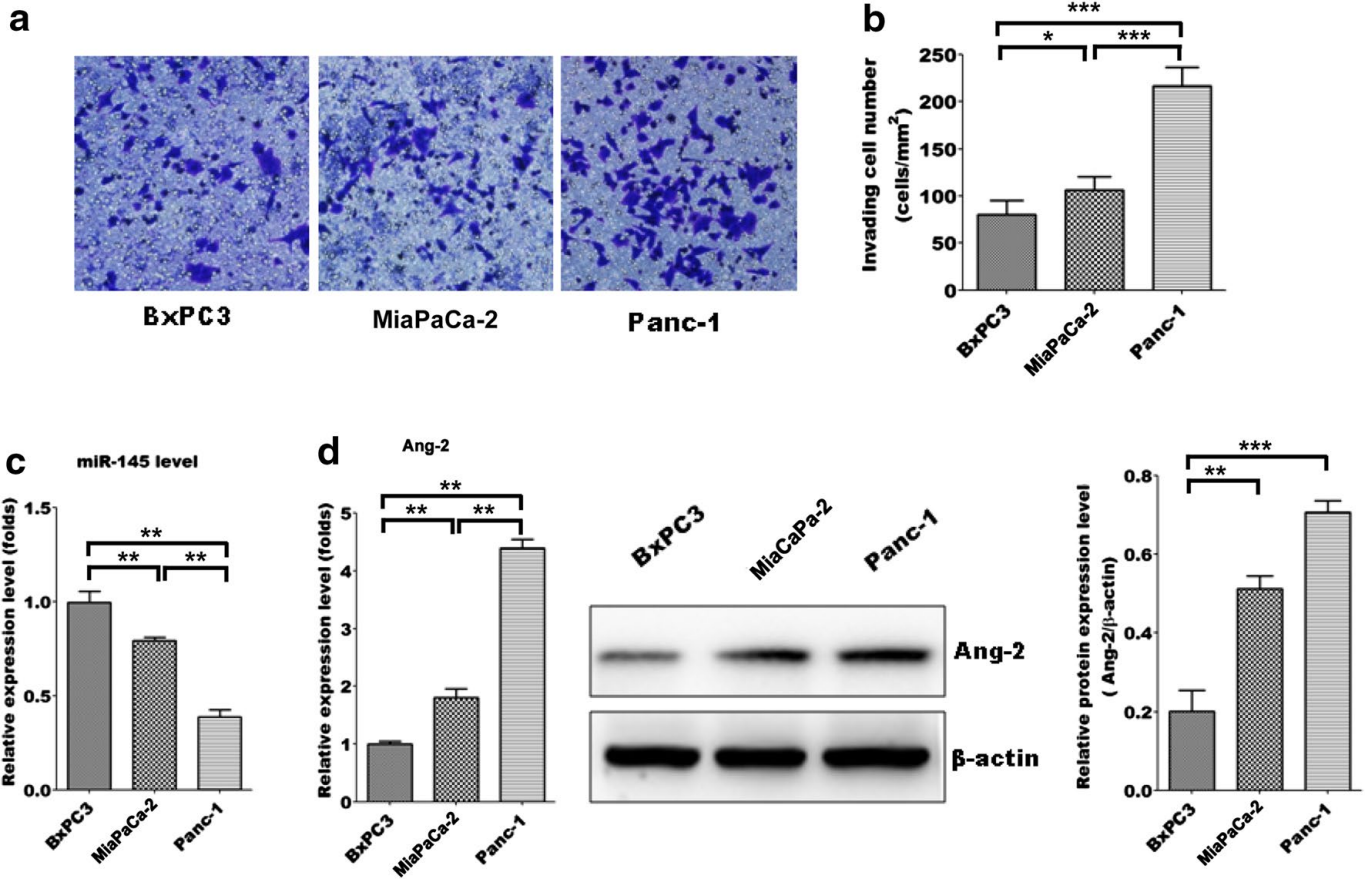
Down-regulation of miR-145 accompanied with up-regulation of Ang-2 in more aggressive pancreatic cancer cells. **a**, **b** The invasion ability of BxPC3, MiaPaca-2 and Panc-1 cells was measured by in vitro invasion assay; **c** the expression level of miR-145 in BxPC3, MiaPaca-2 and Panc-1 cells was measured by qRT-PCR; **d** the expression levels of Ang-2 mRNA and protein in BxPC3, MiaPaca-2 and Panc-1 cells were measured by qRT-PCR and western blotting, respectively. Data represents the mean ± SEM, n = 3, significant differences compared to BxPC3 group are indicated as *P < 0.05, **P < 0.01, ***P < 0.001 (one-way ANOVA followed by Bonferroni test)

To elucidate whether the expression level of miR-145 are correlated with the cell invasion ability of pancreatic cancer, three well-studied human pancreatic cancer cell lines, BxPC3, MiaPaCa-2 and Panc-1 were investigated in the present study. In vitro invasion assay results showed that MiaPaCa-2 and Panc-1 were more aggressive than BxPC3, and our qRT-PCR results demonstrated that the expression levels of miR-145 were lower in MiaPaCa-2 and Panc-1 cells when compared to that in BxPC3 cells (Fig. 1a, b). Moreover, qRT-PCR and western blotting analysis showed that the mRNA and protein levels of anigopoietin-2 were also found to be higher in both Panc-1 and MiaPaCa-2 when compared to that in less invasive BxPC3 cells (Fig. 1d). These findings indicates that the expression of miR-145 and Ang-2 might be correlated with the invasive capacity of pancreatic cancer cells.

### MiR-145 suppressed cell invasion via decreasing the expression of Ang-2 in vitro

**Fig. 2:**
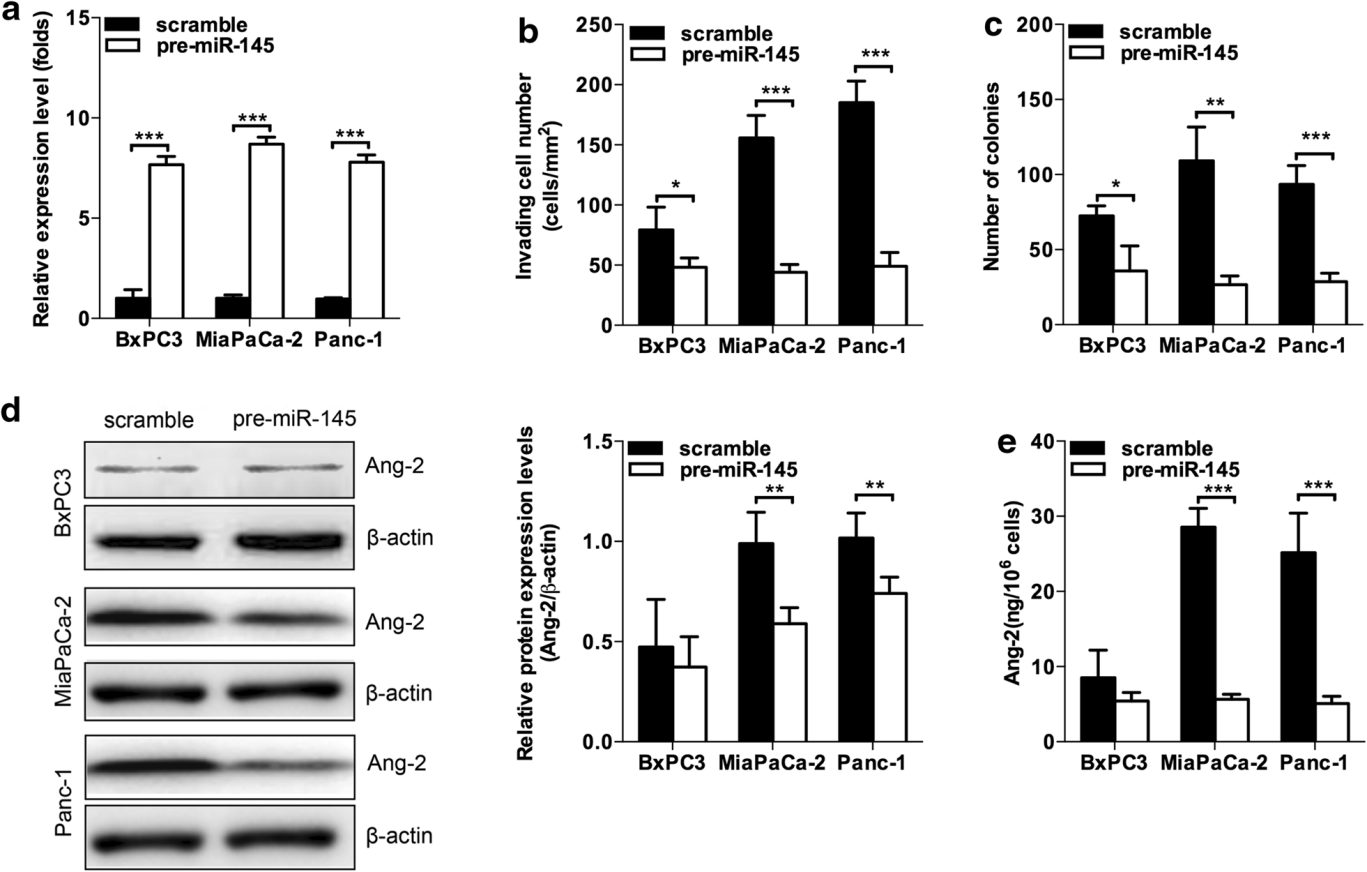
MiR-145 suppressed cell invasion and colony formation via decreasing the expression of Ang-2 in vitro. **a** Ectopic expression of miR-145 increased expression levels of miR-145 in BxPC3, MiaPaCa-2 and Panc-1 cells; **b**, **c** ectopic expression of miR-145 decreased the cell invasion and colony formation ability of BxPC3, MiaPaCa-2 and Panc-1 cells; **d** overexpression of miR-145 decreased Ang-2 protein levels in MiaPaCa-2 and Panc-1 cells but not in BxPC3 cells; **e** the expression of Ang-2 was lower in the supernatant of culture media of miR-145 over-expressing MiaPaCa-2 and Panc-1 cells when compared to control groups. Data represents the mean ± SEM, n = 3, significant differences between groups are indicated as *P < 0.05, **P < 0.01, ***P < 0.001 (unpaired t test)

The role of miR-145 in pancreatic cancer cells was further investigated by using miR-145 gain-of-function study in the human pancreatic cancer cell lines. The results shown that ectopic expressed miR-145 in BxPC3, MiaPaCa-2 and Panc-1 cells (Fig. 2a) significantly decreased thecell invasion and colony formation ability (Fig. 2b, c). Moreover, western blotting results showed that lower protein expression levels of Ang-2 were found in miR-145-overexpressing Panc-1 and MiaPaCa-2 cells, but not in BxPC3 cells when compared with control group transfected with scramble miRNA (Fig. 2d). In addition, down-regulation of Ang-2 were also found in the supernatant of culture media of miR-145-overexpressing Panc-1 and MiaPaCa-2 cells, but not in BxPC3 cells (Fig. 2e). These findings implicated that down-regulation of miR-145 might enhance the in vitro cell invasion and colony formation of pancreatic cancer cells via enhancing the expression of Ang-2.

### MiR-145 inhibited tumor growth and angiogenesis in vivo

**Fig. 3:**
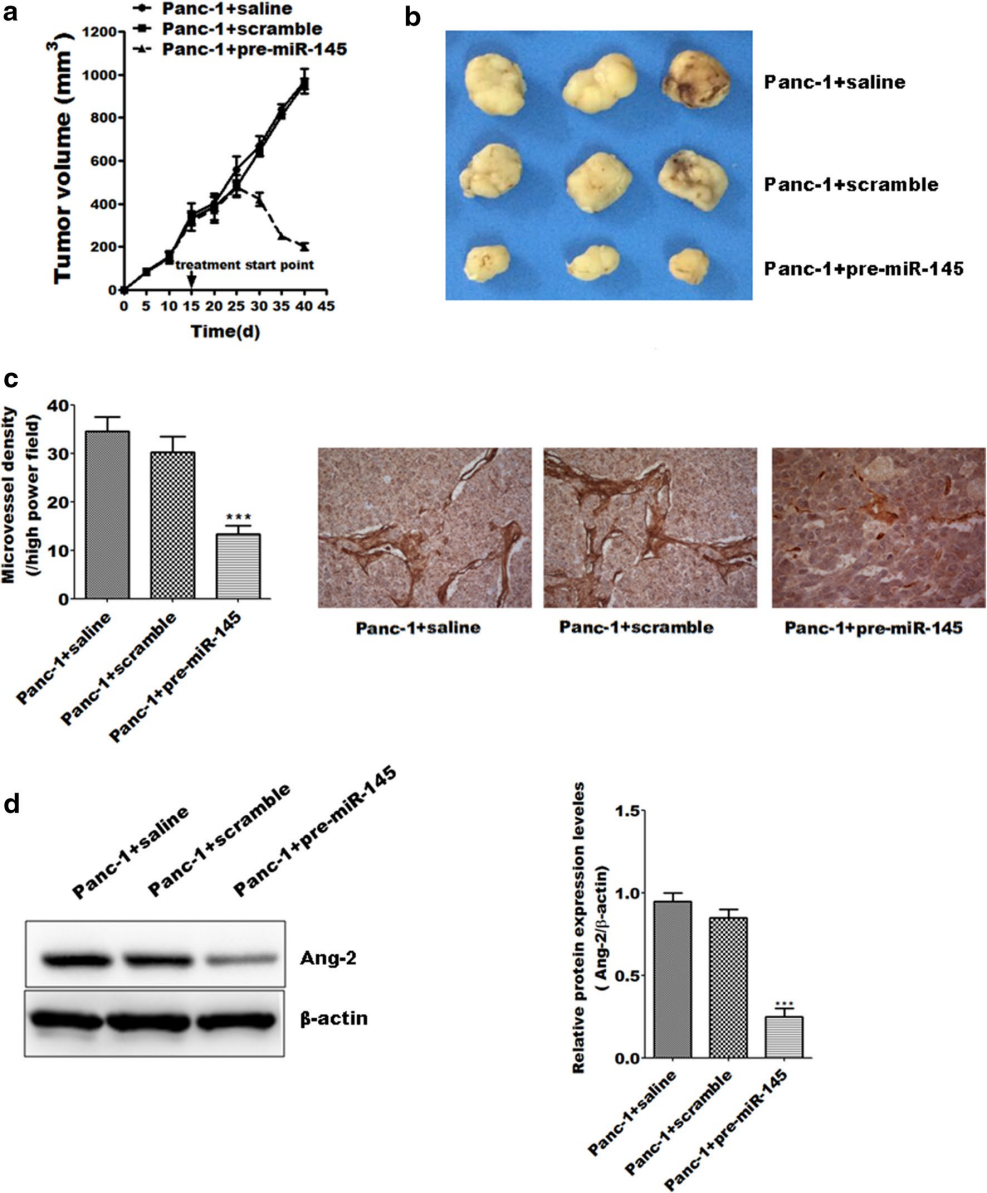
MiR-145 inhibited tumor growth and angiogenesis in vivo. **a**, **b** MiR-145 significantly inhibited the growth of xenografts formed by Panc-1 when compared with saline or vector control group; **c** miR-145 treatment decreased microvessels density in xenografts isolated 45 days after subcutaneous injection when compared to saline or vector control group; **d** the expression levels of Ang-2 in xenografts treated with miR-145 were lower than that in the saline or vector control group. Data represents the mean ± SEM, n = 8, significant differences between groups are indicated as ***P < 0.001 (one-way ANOVA followed by Bonferroni test)

To further elucidate the significance of miR-145 in the tumor growth capacity of pancreatic cancer in vivo, miR-145 were delivered intratumorally in xenografts formed by relative more invasive pancreatic cancer cell line Panc-1. The results showed that treatment of miR-145 significantly inhibited the growth of xenografts formed by Panc-1 when compared to that treated with saline or vector control (Fig. 3a, b). Moreover, MiR-145 treatment also decreased microvessels density as well as expression levels of Ang-2 in xenografts when compared to that treated with saline or vector control (Fig. 3c, d). These findings indicate that miR-145 might inhibit cell growth of pancreatic cancer cells via its anti-angiogenesis effect mediated by down-regulation of Ang-2.

### MiR-145 directly regulated the expression of Ang-2 in pancreatic cancer cells

**Fig. 4:**
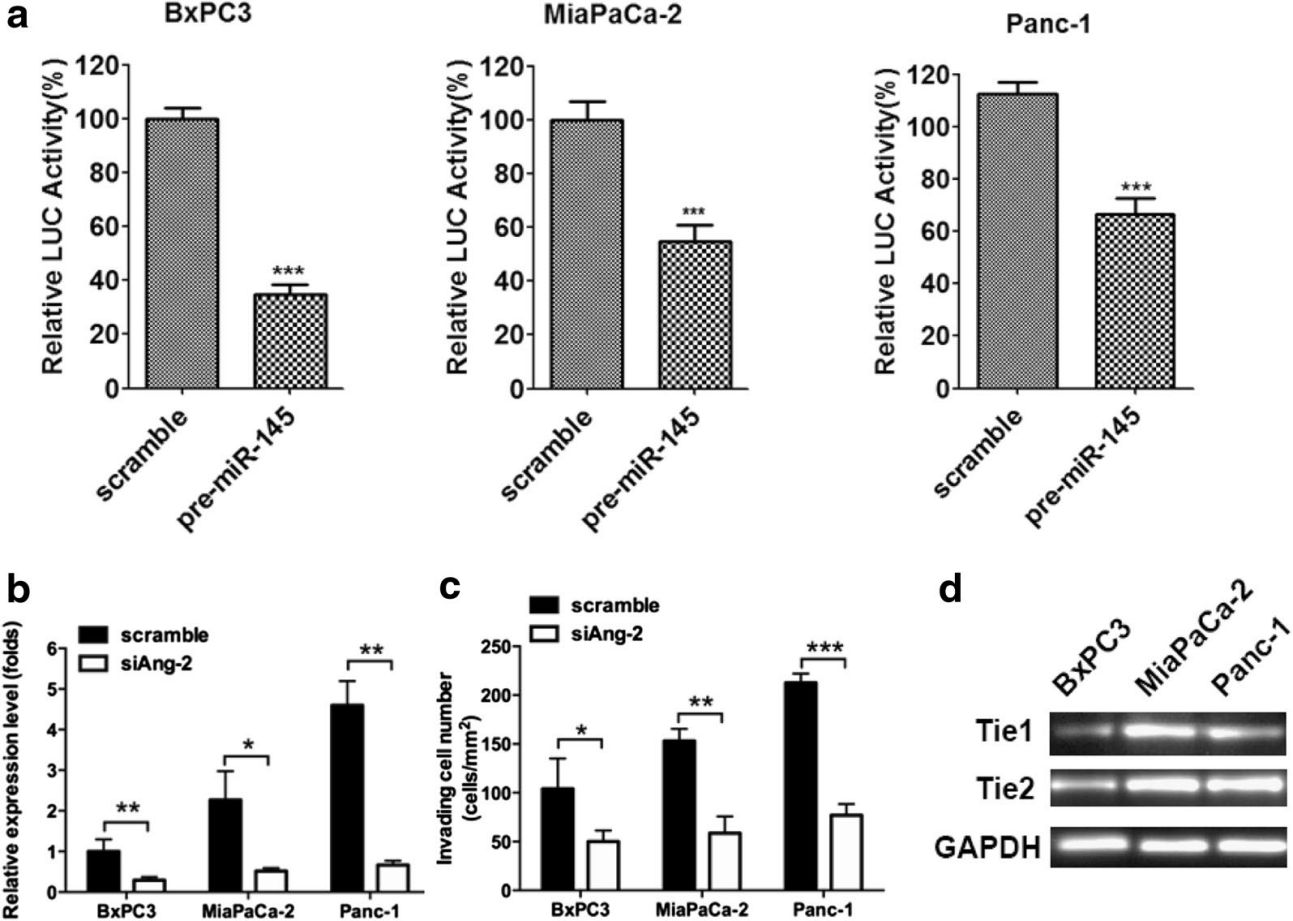
MiR-145 directly regulated the expression of Ang-2 in pancreatic cancer cells. **a** The effect of miR-145 on the luciferase activity of Ang-2 promoter were measured, the luciferase activity of Ang-2 promoter were all decreased in BxPC3, MiaPaCa-2 and Panc-1 cells; **b**, **c** down-regulation of Ang-2 by treatment with siAng-2 decreased the invasion and colony formation ability of BxPC3, MiaPaCa-2 and Panc-1 cells; **d** the expression of Tie1 and Tie2 in BxPC3, MiaPaCa-2 and Panc-1 was determined by reverse transcription PCR. Data represents the mean ± SEM, n = 3, significant differences between groups are indicated as *P < 0.05, **P < 0.01, ***P < 0.001 (unpaired t test)

To further elucidate whether miR-145 could directly regulate the expression of Ang-2 in pancreatic cancer cells, the effect of miR-145 on the luciferase activity of Ang-2 gene 3′-UTR were investigated. The luciferase report assay demonstrated that transiently transfected with pre-miR-145 decreased the luciferase activity of Ang-2 3′-UTR in BxPC3, MiaPaCa-2 and Panc-1 cells (Fig. 4a). Transfection with siAng-2 in BxPC3, MiaPaCa-2 and Panc-1 cells significantly decreased cell invasion and colony formation ability (Fig. 4b, c). Further reverse transcription PCR results demonstrated that Tie1 and Tie2 are presented in BxPC3, MiaPaCa-2 and Panc-1 cells. These findings indicate that miR-145 might regulate the expression of Ang-2 in pancreatic cancer cells directly.

## Discussion

Antiangiogenic therapy targeting the vascular endothelial growth factor (VEGF) pathway has been considered as a standard cancer therapy strategy in the past decade [22, 23]. Ang-2-targeting therapies are now regarded as second-generation antiangiogenic drugs that combine with anti-VEGF to improve the hitherto limited clinical efficacy of established antiangiogenic therapy [24–26]. Recently, Ang-2 antibody treatment combines well with low-dose metronomic chemotherapy showed the effective anti-inflammatory and anti-angiogenic response of endothelial cells [27], provided a potential target of antiangiogenic drug development. We here reported that miR-145 suppressed the cell invasion via directly regulating the expression of Ang-2 in pancreatic cancer cells and ectopic expression of miR-145 inhibited tumor growth and angiogenesis in vivo. This study not only provided the information that Ang-2 may involve in pancreatic cancer cell invasion for the first time, but also provided a new strategy to develop antiangiogenic drug by targeting on miR-145.

The role of miR-145 in cancer have been extensively studied in various types of cancers. Most of the studies were focused on effect of miR-145 on cancer cell proliferation and metastasis. However, its role in tumor angiogenesis remains poorly defined. One study demonstrated miR-145 inhibited tumor angiogenesis and growth by neuroblastoma RAS and VEGF in breast cancers [28]. The role of miR-145 on tumor angiogenesis is also found in osteosarcoma cells as well as colon cancer cells by targeting on VEGF and p70S6K1, respectively [12, 29]. Our study was consistent with previous studies showing the tumor suppressive roles in pancreatic cancer.

In the present study, we demonstrated that miR-145 suppressed cell invasion via directly regulating the expression of Ang-2 in pancreatic cancer. Ang-2 has been found to play an important role in angiogenesis and tumor progression. However, we also found that the mRNA expression level of Ang-2 in Panc-1 cells was significantly higher than that in MiaPaCa-2 cells; though there was no significant difference in Ang-2 protein levels between MiaPaCa-2 and Panc-1 cells. The possible reason could be that the difference of Ang-2 protein levels was very small and the semi-quantitative western blotting assay was not sensitive enough to detect the significant difference; and more sensitive assay be required in the future study. Recent study also showed that Ang-2 mediated beta-1-integrin activation as promoter of endothelial destabilization by enhancing β1-integrin-positive elongated matrix adhesions and actin stress fibers [30]. Because of complexity of Ang-2 signaling pathway, its role still requires further investigation [31]. Based on literature research and bioinformatics analysis (TargetScan), Ang-2 was predicted to be a down-stream mediator of miR-145. The present study showed Ang-2 is regulated by miR-145 in pancreatic cancer cells, which has been confirmed by examining the expressing levels of Ang-2 mRNA and protein as well as the luciferase report assay. As miR-145 have multiple targets, whether Ang-2 being specific for pancreatic cancer cell proliferation and invasion is still questionable. In the future study, we may restore the expression levels of Ang-2 to investigate the specific role of Ang-2 on tumor progression in pancreatic cancer. In addition, Ang-2 exerting its effect by interacting with Tie 1/2 receptor [32], but there is no evidence showing the existence of Tie 1/2 receptor in the pancreatic cancer cells. Therefore, it is necessary for us to determine the distribution and expression of these receptors in the future study, which may help us further understand the mechanistic action of Ang-2 in pancreatic cancer.

The study of miRNA-based therapies is still in its infancy. The first miRNA-based therapy specifically for cancer is using miRNA mimics or miRNA inhibitor, such as using synthetic miR-34a mimic loaded in liposomal nanoparticles to suppress liver cancer [33]. Till now, the most advanced miRNA trial involves use of anti-miR-122 for hepatitis C therapy [34], which can reduce miR-122 expression by complementary binding to miR-122 sequence to lock nucleic acid structure. Meanwhile, several studies have focused on small-molecule compound’s modulation on microRNA expression. So far, the small-molecule modulators of miR-21, miR-122 and miR-34a have been identified with potent biologic activities [6, 35, 36].

In conclusion, our in vitro and in vivo studies suggest that miR-145 had a tumor suppressive effect on the pancreatic cancer cells. Therefore, miR-145 mimics or the small molecular modulators of miR-145 may provide the promising strategy to explore Ang-2 targeting antiangiogenic drugs in the future.

## References

[1] Siegel R, Naishadham D, Jemal A (2013). Cancer statistics, 2013. CA Cancer J Clin.

[2] Hezel AF (2006). Genetics and biology of pancreatic ductal adenocarcinoma. Genes Dev.

[3] Bartel DP (2004). MicroRNAs: genomics, biogenesis, mechanism, and function. Cell.

[4] He L, Hannon GJ (2004). MicroRNAs: small RNAs with a big role in gene regulation. Nat Rev Genet.

[5] Zamore PD, Haley B (2005). Ribo-gnome: the big world of small RNAs. Science.

[6] Xiao Z (2014). A small-molecule modulator of the tumor-suppressor miR34a inhibits the growth of hepatocellular carcinoma. Cancer Res.

[7] Yang XW (2014). miR-145 suppresses cell invasion in hepatocellular carcinoma cells: miR-145 targets ADAM17. Hepatol Res.

[8] Yu CC (2013). miR145 targets the SOX9/ADAM17 axis to inhibit tumor-initiating cells and IL-6-mediated paracrine effects in head and neck cancer. Cancer Res.

[9] Xu N (2009). MicroRNA-145 regulates OCT4, SOX2, and KLF4 and represses pluripotency in human embryonic stem cells. Cell.

[10] Boufraqech M (2014). miR-145 suppresses thyroid cancer growth and metastasis and targets AKT3. Endocr Relat Cancer.

[11] Sachdeva M, Mo YY (2010). MicroRNA-145 suppresses cell invasion and metastasis by directly targeting mucin 1. Cancer Res.

[12] Xu Q (2012). MiR-145 directly targets p70S6K1 in cancer cells to inhibit tumor growth and angiogenesis. Nucleic Acids Res.

[13] Zhu Z (2014). MicroRNA-145 directly targets the insulin-like growth factor receptor I in human bladder cancer cells. FEBS Lett.

[14] Kavanagh T (2010). Process evaluation of appreciative inquiry to translate pain management evidence into pediatric nursing practice. Implement Sci.

[15] Gregersen LH (2010). MicroRNA-145 targets YES and STAT1 in colon cancer cells. PLoS One.

[16] Khan S (2014). MicroRNA-145 targets MUC13 and suppresses growth and invasion of pancreatic cancer. Oncotarget.

[17] Hu B, Cheng SY (2009). Angiopoietin-2: development of inhibitors for cancer therapy. Curr Oncol Rep.

[18] Lewis CE, De Palma M, Naldini L (2007). Tie2-expressing monocytes and tumor angiogenesis: regulation by hypoxia and angiopoietin-2. Cancer Res.

[19] Zhou J (2011). Anti-angiogenesis by lentivirus-mediated small interfering RNA silencing of angiopoietin-2 gene in pancreatic carcinoma. Technol Cancer Res Treat.

[20] Zhang ZX (2013). Knockdown of angiopoietin-2 suppresses metastasis in human pancreatic carcinoma by reduced matrix metalloproteinase-2. Mol Biotechnol.

[21] Xiao Z (2014). Role of microRNA-95 in the anticancer activity of Brucein D in hepatocellular carcinoma. Eur J Pharmacol.

[22] Carmeliet P, Jain RK (2011). Molecular mechanisms and clinical applications of angiogenesis. Nature.

[23] Ferrara N, Kerbel RS (2005). Angiogenesis as a therapeutic target. Nature.

[24] Holopainen T (2012). Effects of angiopoietin-2-blocking antibody on endothelial cell-cell junctions and lung metastasis. J Natl Cancer Inst.

[25] Kienast Y (2013). Ang-2-VEGF-A CrossMab, a novel bispecific human IgG1 antibody blocking VEGF-A and Ang-2 functions simultaneously, mediates potent antitumor, antiangiogenic, and antimetastatic efficacy. Clin Cancer Res.

[26] Koh YJ (2010). Double antiangiogenic protein, DAAP, targeting VEGF-A and angiopoietins in tumor angiogenesis, metastasis, and vascular leakage. Cancer Cell.

[27] Srivastava K (2014). Postsurgical adjuvant tumor therapy by combining anti-angiopoietin-2 and metronomic chemotherapy limits metastatic growth. Cancer Cell.

[28] Zou C (2012). MiR-145 inhibits tumor angiogenesis and growth by N-RAS and VEGF. Cell Cycle.

[29] Fan L (2012). MicroRNA-145 targets vascular endothelial growth factor and inhibits invasion and metastasis of osteosarcoma cells. Acta Biochim Biophys Sin (Shanghai).

[30] Hakanpaa L (2015). Endothelial destabilization by angiopoietin-2 via integrin beta1 activation. Nat Commun.

[31] Thurston G, Daly C (2012). The complex role of angiopoietin-2 in the angiopoietin-tie signaling pathway. Cold Spring Harb Perspect Med.

[32] Singh N (2014). Angiotensin-(1-7) reverses angiogenic dysfunction in corpus cavernosum by acting on the microvasculature and bone marrow-derived cells in diabetes. J Sex Med.

[33] Bouchie A (2013). First microRNA mimic enters clinic. Nat Biotechnol.

[34] Janssen HL (2013). Treatment of HCV infection by targeting microRNA. N Engl J Med.

[35] Gumireddy K (2008). Small-molecule inhibitors of microrna miR-21 function. Angew Chem Int Ed Engl.

[36] Young DD (2010). Small molecule modifiers of microRNA miR-122 function for the treatment of hepatitis C virus infection and hepatocellular carcinoma. J Am Chem Soc.

